# Morphologic and Functional Connectivity Alterations of Corticostriatal and Default Mode Network in Treatment-Naïve Patients with Obsessive-Compulsive Disorder

**DOI:** 10.1371/journal.pone.0083931

**Published:** 2013-12-16

**Authors:** Jingming Hou, Lingheng Song, Wei Zhang, Wenjing Wu, Jian Wang, Daiquan Zhou, Wei Qu, Junwei Guo, Shanshan Gu, Mei He, Bing Xie, Haitao Li

**Affiliations:** 1 Department of Radiology, Southwest Hospital, Third Military Medical University, Chongqing, China; 2 Department of Clinical Psychology, Southwest Hospital, Third Military Medical University, Chongqing, China; Beijing Normal University,Beijing, China

## Abstract

**Background:**

Previous studies have demonstrated that structural deficits and functional connectivity imbalances might underlie the pathophysiology of obsessive-compulsive disorder (OCD). The purpose of the present study was to investigate gray matter deficits and abnormal resting-state networks in patients with OCD and further investigate the association between the anatomic and functional alterations and clinical symptoms.

**Methods:**

Participants were 33 treatment-naïve OCD patients and 33 matched healthy controls. Voxel-based morphometry was used to investigate the regions with gray matter abnormalities and resting-state functional connectivity analysis was further conducted between each gray matter abnormal region and the remaining voxels in the brain.

**Results:**

Compared with healthy controls, patients with OCD showed significantly increased gray matter volume in the left caudate, left thalamus, and posterior cingulate cortex, as well as decreased gray matter volume in the bilateral medial orbitofrontal cortex, left anterior cingulate cortex, and left inferior frontal gyrus. By using the above morphologic deficits areas as seed regions, functional connectivity analysis found abnormal functional integration in the cortical-striatum-thalamic-cortical (CSTC) circuits and default mode network. Subsequent correlation analyses revealed that morphologic deficits in the left thalamus and increased functional connectivity within the CSTC circuits positively correlated with the total Y-BOCS score.

**Conclusion:**

This study provides evidence that morphologic and functional alterations are seen in CSTC circuits and default mode network in treatment-naïve OCD patients. The association between symptom severity and the CSTC circuits suggests that anatomic and functional alterations in CSTC circuits are especially important in the pathophysiology of OCD.

## Introduction

Obsessive-compulsive disorder (OCD), a common and disabling neuropsychiatric disorder, affects 2%–3% of the general population [1]. OCD is characterized by persistent intrusive thoughts or images (obsessions) and/or a strong desire to perform certain actions or activities (compulsions). Despite its high morbidity, the underlying pathophysiology of OCD is unclear. 

Previous neuropsychological and animal studies have shown that abnormalities of cortical-striatal-thalamic-cortical (CSTC) circuits may play a key role in the pathogenesis of OCD [2-5]. Although the findings of structural and functional neuroimaging studies have been relatively consistent with this view, findings from these studies have been inconsistent [6-9]. For example, the striatum, as a crucial region in CSTC circuits, was reported to have structural and functional alterations [10-13], or no alterations [14-17]. Confounders associated with psychotropic medication exposure, duration of illness, relatively small sample sizes, and methodological differences between studies may contribute to the inconsistency across studies. Several studies have shown that brain structure and function can be significantly influenced by psychotropic medication [18,19]. Previous some neuroimaging studies may have detected effects of psychotropic medication or illness chronicity rather than direct illness effects on brain structure and function. In this context, we considered it especially useful to conduct a morphologic and functional study to explore the core pathophysiology of OCD in treatment-naïve OCD patients. 

There are increasing functional studies which have provided strong evidence that the pathophysiology of OCD is unlikely to be the result of a single abnormal brain region or neurotransmitter system. Instead, it could be conceptualized as a distributed neuronal brain network [20,21]. Therefore, brain abnormalities in OCD are much more likely to be present in functional connectivity between brain regions, rather than within discrete brain regions [22-25]. Several fMRI studies have reported functional connectivity aberrancies within CSTC circuits during resting-state [11,24-27]. The CSTC circuits have extensive connectivity to many cortical and subcortical regions and are considered to play a role in the executive function, cognitive and behavioral regulation, and conflict monitoring [28-30]. The dysregulation of these connectivities within CSTC circuits may be related to OCD patients’ impaired executive performance [31], inability to inhibit cognitions and behaviors [32], and enhanced error monitoring processes [33,34]. In addition to functional disturbances in CSTC circuits, recent studies also have shown that the function of the default mode network (DMN) was disrupted in OCD patients compared with healthy controls [22,23]. The DMN is a prominent large-scale brain network that preferentially activates when individuals engage in internal tasks such as episodic memory retrieval, mental imagery, inner speech, and planning of future events. In humans, the function of DMN has been hypothesized to generate spontaneous thoughts during mind-wandering [35], and dysfunction of DMN has been considered to be associated with some OCD clinical symptoms such as the inability to get rid of persistent intrusive thoughts and images [22]. In summary, all of these resting-state functional studies in OCD provided evidence for abnormal functional organization during resting-state and enhanced our understanding of the psychopathology of OCD.

In the current study, we combined voxel-based morphometry (VBM) and resting-state functional connectivity analysis in order to perform a comprehensive evaluation of the neural circuitry of OCD. Along these lines, the main objective of this study was to (1) identify brain regions with gray matter abnormalities in first-episode treatment-naïve patients with OCD, (2) investigate the brain functional connectivity using the observed gray matter abnormalities areas as seed regions, and (3) explore the clinical significance of structural deficits and functional connectivity by focusing on their association with symptom severity and disease duration in OCD patients.

## Methods and Materials

### Participants

The medical ethics committee of Third Military Medical University (Chongqing, China) approved the current study and all participants gave written informed consent to the participation according to the Declaration of Helsinki. Thirty-four patients who were seeing a doctor for the first time and met the DSM-IV criteria for OCD were recruited for this study from the Department of Clinical Psychology of the Southwest Hospital between September 2010 and December 2012. Diagnosis of OCD and duration of illness were determined by two qualified psychiatrists (Dr W. Qu and Dr J. Guo) using the Structured Clinical Interview according to the DSM-IV criteria (SCID). The severity of OCD symptoms was assessed using the Yale-Brown Obsessive-Compulsive Scale (Y-BOCS) [36,37]. In addition, the 17-item Hamilton Depression Rating Scale (HDRS) and 14-item Hamilton Anxiety Rating Scale (HARS) were administered to evaluate the severity of depression and anxiety symptoms, respectively [38,39]. No patient in this cohort met the criteria for major depressive disorder or Tourette syndrome.

Thirty-four healthy controls were recruited among hospital staff and by advertisements on the internet. All controls were screened by using the SCID-Nonpatient Edition to ascertain that there was no personal history of psychiatric and neurologic illnesses. The controls were also interviewed to confirm the lifetime absence of psychiatric diseases in their first-degree relatives. 

Exclusion criteria for OCD patients and healthy controls were as follows: a history of other psychiatric or neurological illness; a history of drug or alcohol abuse; pregnancy; serious physical illness; and contraindications to MR scanning. Conventional MR images (T2-weighted images) were inspected by two experienced neuroradiologists (Dr J. Wang and Dr H. Li) to exclude gross abnormalities. Co-morbid depressive and anxious symptoms were not considered as an exclusion criterion, if OCD was the primary clinical diagnosis. Of the original participants, one female patient and one male healthy control were excluded from the final analysis; the patient was excluded because of excessive head motion during MR scanning (>2 mm in translation) and the control was excluded owing to an incidental finding on conventional MR images (intracranial arachnoid cyst). The final study consisted of 33 OCD patients and 33 healthy controls. The two groups were well-matched for age, gender, and years of education. All participants were right-handed. Clinical and demographic data from all 66 participants are shown in Table 1. 

**Table 1:** Demographic and clinical characteristics of OCD patients and healthy controls.

Characteristics	OCD patients(n=33)	controls(n=33)	P value
Gender (male: female)	18:15	18:15	1
Mean age (years)	25.3±9.6	25.0±9.1	0.88
Educational level (years)	12.0±3.6	12.8±3.8	0.65
Illness duration (years)	6.3±9.9	_	_
HARS Total score	6.3±3.2	_	_
HDRS Total score	9.1±3.5	_	_
Y-BOCS Total score	21.1±6.3	_	_
Obsessive subscale score	11.3±3.6	_	_
Compulsive subscale score	9.8±3.9	_	_

Data are expressed as the mean ± SD, SD: standard deviation; OCD: obsessive-compulsive disorder; HARS: Hamilton Anxiety Rating Scale; HDRS: Hamilton Depression Rating Scale; Y-BOCS, Yale-brown obsessive-compulsive scale.

### MRI data acquisition

All structural and functional images were obtained using a 3.0 T MRI system (TIM Trio, Siemens, Erlangen, Germany) with an eight-channel phased array head coil. During the MRI scans, all participants were instructed to remain relaxed with their eyes closed and lie still without moving. The resting-state functional images were acquired using an echo-planar-imaging (EPI) sequence: 36 axial slices with a slice thickness of 4 mm and no slice gap; repetition time, 2000 ms; echo time, 30 ms; flip angle, 90°; field of view, 256 × 256 mm^2^; matrix, 64 × 64; and isotropic voxel, 4 × 4 × 4 mm^3^. For each participant, the fMRI scanning lasted for 480 s and 240 volumes were obtained. In addition, a high resolution structural T1-weighted anatomic sequence was acquired in a sagittal orientation using a 3-dimensional magnetization-prepared rapid gradient-echo (3D MP-RAGE) sequence, as follows: repetition time, 1900 ms; echo time, 2.52 ms; flip angle, 15°; slice thickness, 1 mm; matrix, 256 × 256; and isotropic voxel, 1 × 1 × 1 mm^3^.

### Voxel-Based Morphometric Analysis

The voxel-based morphometric analysis was performed using SPM8 software (Statistical Parametric Mapping; http://www.fil.ion.ucl.ac.uk/spm/) implemented in MATLAB 2010b (Math Works, Natick, MA, USA). First, all structural MR images were manually reoriented to place the anterior commissure at the origin of the 3D MNI space. Then, the structural images were segmented into gray matter, white matter and cerebrospinal fluid using the unified standard segmentation option in SPM8. After segmentation, the gray matter template for a group of individuals was generated from the entire image dataset using the Diffeomorphic Anatomical Registration Through Exponentiated Lie algebra (DARTEL) toolbox following John Ashburner’s chapter in the standard version [40]. Next, the resulting images were spatially normalized into the MNI space using affine spatial normalization. The total amount of gray matter volume of each voxel was obtained through modulation by multiplying the gray matter concentration map by the non-linear determinants derived from the spatial normalization step. Finally, the resulting gray matter images were then smoothed with an isotropic Gaussian kernel (full-width half-maximum=8 mm). 

Voxel-wise comparisons of gray matter volume between the OCD and control groups were performed using two-sample *t*-tests. Age, gender, and total intracranial volume were modeled as covariates of no interest. The statistical significance of group differences in each region was set at a *p*<0.05 with family-wise error correction. To identify the association between gray matter abnormalities and clinical characteristics including Y-BOCS score and disease duration, the average values of gray matter volume for all the voxels in abnormal areas revealed by VBM analysis, were extracted and correlated with the Y-BOCS score and disease duration using Pearson correlation analysis.

### Functional Connectivity Analysis

Functional connectivity preprocessing and statistical analysis were also done with SPM8 software. To avoid manipulation error confounds and standardize the process, we used the batch-processing tool data processing assistant for resting-state fMRI (DPARSF; http://www.restfmri.net) [41]. For the resting state fMRI data of each subject, the first 10 volumes of functional images were discarded to allow for steady-state magnetization and stabilization of participant status. EPI images were slice-timing corrected to the middle slice acquired in time, and realigned and resliced to correct for head motion with a mean volume created. Structural images were co-registered with the mean volume of functional images and subsequently segmented by an inbuilt unified segmentation routine in SPM8. The parameter created by segmentation was then applied to functional images, non-linear normalization to the MNI template brain, and each voxel was resampled to isotropic 3 mm × 3 mm × 3 mm. As a final step, the resting state fMRI images were smoothed using a 8-8-8 mm FWHM Gaussian kernel. The head motion of all participants during resting-state fMRI acquisition was observed, and data were discarded if the translation exceeded 2 mm or if rotation exceeded 2°.

Functional connectivity was analyzed using the REST software package (http://www.restfmri.net) using a seed voxel correlation approach [42]. Because structural MRI exhibited gray matter alteration in OCD patients, we tested whether or not the intrinsic connectivity of these areas had been affected. The gray matter deficits areas that resulted from voxel-based morphometric analysis were utilized as regions of interest (ROIs). Several possible spurious sources of variances, including the estimated head motion parameters, global brain average signals, and average signals from the cerebrospinal fluid and white matter, were removed from the data through linear regression. After bandpass filtering (0.01-0.08 Hz) and linear trend removal, a reference time series for each seed was extracted by averaging the time series of voxels within each ROI. A correlation analysis was conducted between the seed ROI and the remaining voxels in the whole brain. The resulting *r* values were converted using Fisher’s *r*-to-*z* transformation to improve the Gaussianity of their distribution.

To compare the difference in functional connectivity between the OCD and healthy control group, two-sample *t*-tests were used. Age, gender, years of education and total gray matter volume were entered as co-variates of no interest. The significance level of group differences was set at a *p*<0.05 with multiple comparison corrected by AlphaSim methods (combined height threshold of a *p*<0.001 and a minimum cluster size of 22 voxels). Subsequently, the group differences were correlated with the total Y-BOCS score and disease duration using Pearson correlation analysis.

### Validations: Reproducibility

To further evaluate the reproducibility of our findings, we conducted the following procedures.

### The effects of without global signal regression

In the preprocessing of resting state fMRI data, there is much debate in the community about the use of global signal regression [42-45]. Although the global signal regression could reduce the effect of the physiological noise and improve the specificity of positive correlations, it also may generate artificial negative correlations and thus alters intrinsic correlation structure of the brain networks [46]. Notably, a recent fMRI study suggested that global signal regression can induce major bias into the analysis of group differences [45]. To explore the reproducibility of our results, in this study, we reanalyzed our data without regressing out global signal.

### The effects of structural gray matter alteration

Previous studies have shown that that resting-state brain function may be affected by structural gray matter alteration [47]. In this study, we found gray matter alteration in multiple regions in OCD patients. To reduce the effect of brain structural alteration on resting-state functional connectivity measurements, we reanalyzed our data with gray matter volume as an extra covariate in the statistical models.

## Results

### Morphometric Analysis

**Figure 1:**
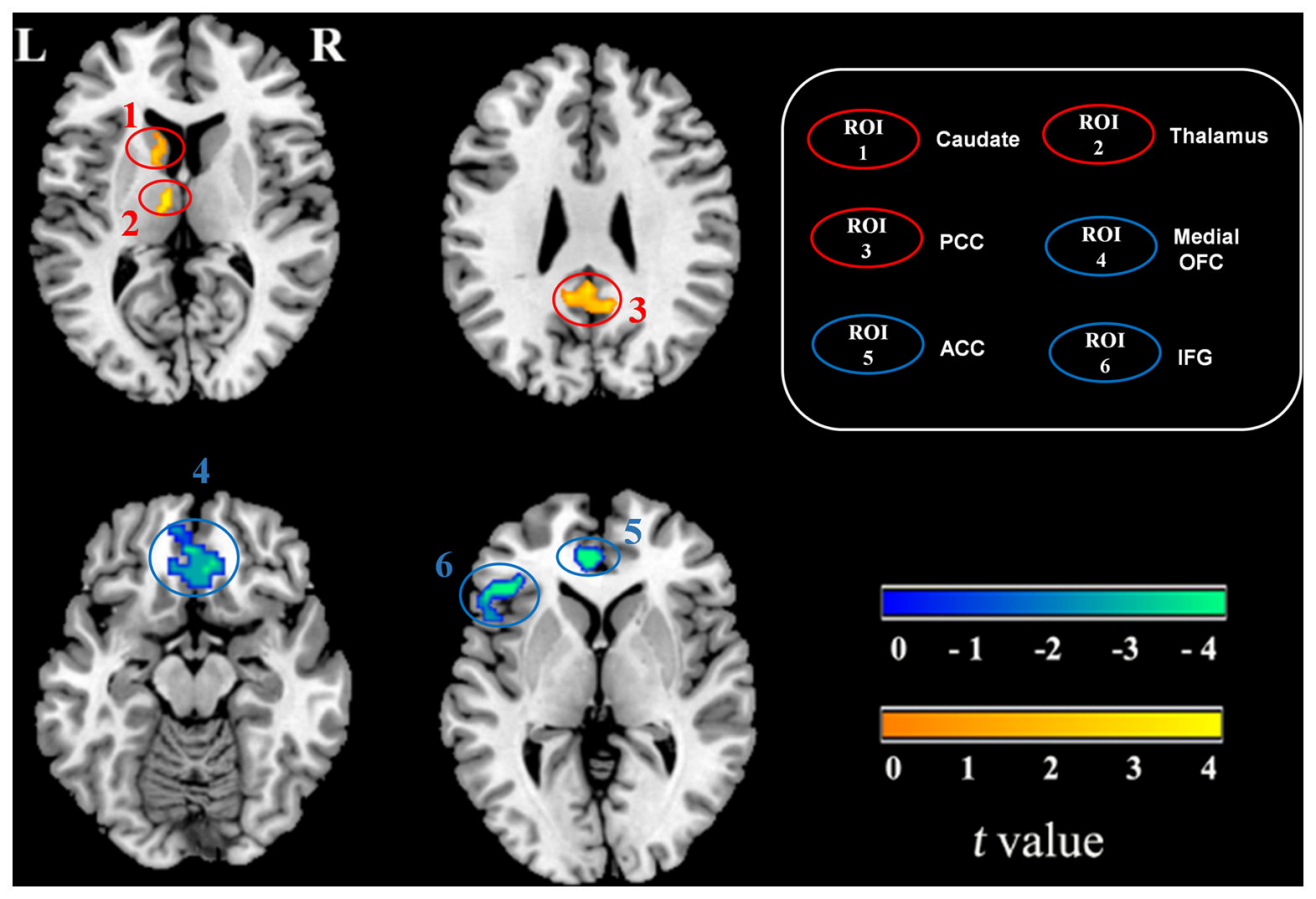
Gray matter difference between OCD patients and healthy controls. The warm color denotes the brain regions having increased gray matter volume, and the cold color denotes the brain regions having reduced gray matter volume in OCD patients. Maps threshold were set at *p*<0.05 with family-wise error correction.

There were no significant differences (*p* = 0.61) in the total gray matter volume between first-episode OCD patients (609.98±64.32 ml) and healthy controls (615.63±60.95 ml). Compared with healthy controls, OCD patients showed significantly increased regional gray matter volume mainly in the left caudate (MNI_xyz_= -12, 19, and 9, respectively; Cluster size = 29 voxels), left thalamus (MNI_xyz_= -6, -12, and 9; Cluster size = 24 voxels) and posterior cingulate cortex (MNI_xyz_= -3, -48, and 30; Cluster size = 51 voxels), as well as decreased gray matter volume in the bilateral medial OFC (MNI_xyz_= 3, 33, and -15; Cluster size = 69 voxels), left ACC (MNI_xyz_= -4, 43, and 3; Cluster size = 27 voxels) and left inferior frontal gyrus (MNI_xyz_= -53, 20, and 3; Cluster size = 36 voxels; Figure 1). 

### Functional Connectivity Analysis

To characterize the alteration of functional networks involving brain areas associated with abnormal gray matter volume, functional connectivity analysis was performed using a seed voxel correlation approach. According to the morphometric analysis results, six brain regions which showed abnormal gray matter volume in OCD patients were selected as seed regions for functional connectivity analysis on resting-state fMRI data; specifically, 3 increased gray matter volume areas (left caudate, left thalamus and posterior cingulate cortex) and 3 decreased gray matter volume areas (medial OFC, left ACC and left inferior frontal gyrus) were selected. There were no significant differences between the two groups in head motion (translation: OCD = 9.86E-2 ± 3.19E-2 mm, controls = 9.63E-2 ± 3.23E-2 mm, *p* = 0.76; rotation: OCD = 1.38E-3 ± 0.53E-3 degree, controls = 1.27E-3 ± 0.49E-3 degree, *p* = 0.53). 

**Figure 2:**
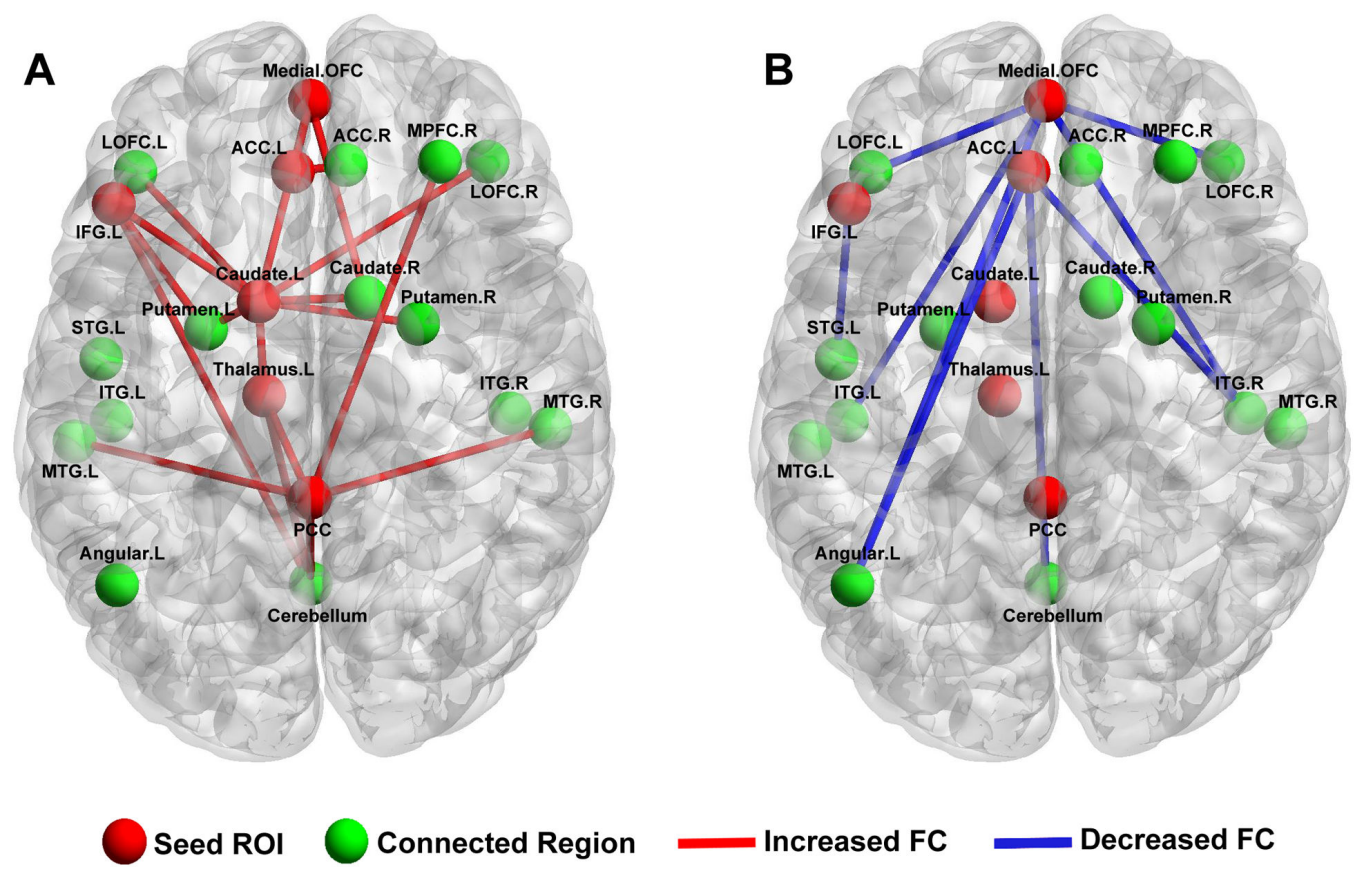
Functional connectivity difference between OCD patients and healthy controls. The red line denotes the brain regions having increased functional connectivity, and the blue line denotes the brain regions having reduced functional connectivity in OCD patients. Maps threshold were set at *p*<0.05 with AlphaSim correction. L, left; R, right; OFC, orbitofrontal cortex; ACC, anterior cingulate cortex; PCC, posterior cingulate cortex. IFG, inferior frontal gyrus; ITG, inferior temporal gyrus; STG, superior temporal gyrus; MTG, middle temporal gyrus. Results are displayed by using the BrainNet Viewer [84] (http://www.nitrc.org/projects/bnv/).

When the seed was located in the left caudate, patients with OCD showed increased functional connectivity mainly in the bilateral lateral OFC, putamen, right caudate, left thalamus, and left inferior frontal gyrus. When the seed was located in the left thalamus, patients with OCD showed increased functional connectivity in the posterior cingulate cortex (PCC), cerebellum, and left caudate, as well as decreased functional connectivity in the right inferior parietal lobe. When the seed was located in the PCC, patients with OCD showed increased functional connectivity in the cerebellum, right medial frontal gyrus, left thalamus, and bilateral middle temporal gyrus. When the seed was located in the medial OFC, patients with OCD showed increased functional connectivity in the bilateral caudate and left ACC, as well as decreased functional connectivity in the bilateral lateral OFC, inferior temporal gyrus, left middle frontal gyrus and left angular. When the seed was located in the left ACC, patients with OCD showed increased functional connectivity in the left caudate, right ACC and medical OFC, as well as decreased functional connectivity in the right inferior temporal gyrus, cerebellum, and left angular. When the seed was located in the left inferior frontal gyrus, patients with OCD showed increased functional connectivity in the left putamen, caudate, and cerebellum, as well as decreased functional connectivity in the left superior temporal gyrus (Figure 2, Table 2).

**Table 2:** Brain regions with significantly altered functional connectivity in patients with OCD.

		MNI coordinate			Voxel	Peak	
Seed ROI	Connected region	x	y	z	size	t value	Direction *
**OCD patients > Healthy controls**							
Left caudate	Left lateral OFC	-40	40	-6	33	3.86	Positive
	Right lateral OFC	30	30	-10	40	3.86	Positive
	Right caudate	15	12	9	74	5.74	Positive
	Left putamen	-22	5	5	59	5.31	Positive
	Right putamen	21	6	5	20	4.08	Positive
	Left IFG	-46	21	3	16	4.39	Positive
	Left thalamus	-24	12	12	19	4.38	Positive
Left thalamus	Cerebellum	6	-54	-45	24	4.06	Negative
	PCC	-6	-39	0	20	4.04	Positive
	Left caudate	-18	-21	-15	21	3.83	Positive
PCC	Cerebellum	-3	-51	-40	42	4.69	Negative
	Right medial frontal gyrus	6	54	41	15	4.79	Positive
	Left middle temporal gyrus	-62	-20	-3	30	3.94	Positive
	Right middle temporal gyrus	57	-33	2	31	3.57	Positive
	Left thalamus	-25	13	10	20	4.33	Positive
Medial OFC	Left caudate	-13	21	0	16	3.96	Positive
	Right caudate	-13	21	-1	15	4.83	Positive
	Left ACC	-4	31	-5	10	4.86	Positive
Left ACC	Left caudate	-6	36	3	27	5.29	Positive
	Right ACC	5	41	3	46	5.33	Positive
	Medial OFC	-2	51	-7	15	5.23	Positive
Left IFG	Left putamen	-24	12	12	45	4.86	Positive
	Left caudate	-24	12	12	30	4.82	Positive
	Cerebellum	33	-54	-51	30	3.86	Negative
**OCD patients < Healthy controls**							
Medial OFC	Left lateral OFC	-23	65	-3	15	-4.84	Positive
	Right lateral OFC	40	60	-3	40	-4.71	Positive
	Left inferior temporal gyrus	-45	5	-34	66	-5.51	Positive
	Right inferior temporal gyrus	49	-12	-36	55	-5.36	Positive
	Left angular	-42	-81	30	67	-4.76	Positive
Left ACC	Right inferior temporal gyrus	45	-15	-36	33	-4.36	Positive
	Left angular	-54	-66	30	15	-3.52	Positive
	Cerebellum	5	-39	-18	13	-5.11	Negative
Left IFG	Left superior temporal gyrus	-42	9	-18	40	-4.72	Both

ROI, region of interest; OCD, obsessive-compulsive disorder; OFC, orbitofrontal cortex; ACC, anterior cingulate cortex; PCC, posterior cingulate cortex; IFG, inferior frontal gyrus. (*p*<0.05, corrected with Alphasim). * Direction of effects in each region: Group differences were driven by differences in positive connectivity in OCD and controls (Positive), negative connectivity in OCD and controls (Negative), or negative connectivity in OCD and positive connectivity in controls (Both).

### Brain-Behavioral Associations

**Figure 3:**
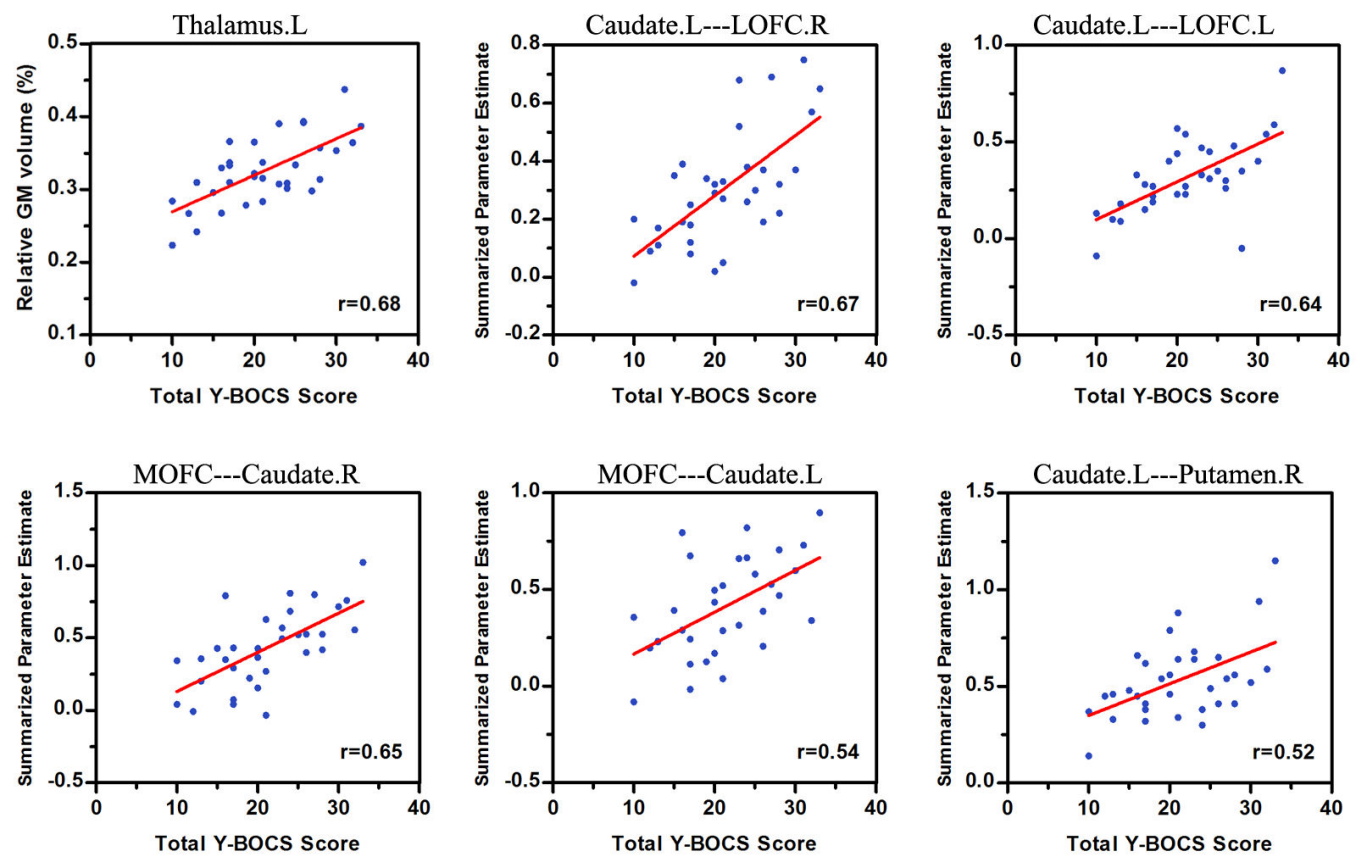
Positive correlation between total Y-BOCS score and brain gray matter volume and functional connectivity strength in abnormal areas. L, left; R, right; LOFC, lateral orbitofrontal cortex; MOFC, medial orbitofrontal cortex.

The average gray matter volume values and the strength of functional connectivity in structural abnormal areas were extracted and correlated with disease duration and overall OCD symptom severity. No significant correlations were found for the gray matter volumes or functional connectivity strength with the disease duration in patients with OCD. Significant positive correlations were observed between the gray matter volumes in the left thalamus and total Y-BOCS scores (*r* = 0.68, *p*<0.001). In addition, significant positive correlations were also found between the total Y-BOCS scores and the strength of the functional connectivity of multiple areas within the cortical-striatum-thalamic circuits. When the seed was located in the left caudate, significant positive correlations were observed between the total Y-BOCS scores and the strength of functional connectivity in the bilateral lateral OFC (left, *r* = 0.67, *p*<0.001; right, *r* = 0.64, *p*<0.001) and right putamen (*r* = 0.52, *p* = 0.002). When the seed was located in the medial OFC, significant positive correlations were observed between the total Y-BOCS scores and the strength of functional connectivity in the bilateral caudate (left, *r* = 0.54, *p* = 0.001; right, *r* = 0.65, *p*<0.001; Figure 3).

### Reproducibility of Our Findings

We found that our main results were reproducible after considering the effects of without global signal removal (Figure S1 and S2) and gray matter alteration (Figure S3). Without regressing the global signal out, we observed that the OCD groups showed functional connectivity alterations in the CSTC circuits and default mode network (Figure S2), which was consistent with those with global signal removal. However, it was noted that without global signal removal, we found more widespread functional connectivity alterations in the CSTC circuits, DMN and some other regions, including the cerebellum and temporal cortex. It indicates that the global signal might be physiologically meaningful. In addition, after taking gray matter volume as extra covariates, the functional connectivity group difference exhibited highly similar spatial patterns with those without gray matter volume correcting (Figure S3). This indicates that functional connectivity alterations observed in the OCD patients can be only partly explained by structural deficits.

## Discussion

The present study demonstrated brain gray matter abnormalities and network alterations in treatment-naïve OCD patients by combining structural MRI and resting-state fMRI techniques. Patients with OCD showed brain gray matter abnormalities primarily in the CSTC circuits and DMN (i.e., medial OFC, left ACC, left caudate, left thalamus, PCC, and left inferior frontal gyrus). Moreover, by using the above morphologic deficits areas as seed regions, functional connectivity analysis revealed abnormal functional integration in the CSTC circuits and default mode network in OCD patients. Subsequent correlation analyses revealed that morphologic deficits in the left thalamus and increased functional connectivity within the cortical-striatum-thalamic circuits positively correlated with the total Y-BOCS score in patients with OCD, providing a link between CSTC circuits connectivity and OCD clinical symptom severity. 

### The voxel-based morphometric analysis

In recent years, many neuroimaging studies have explored the pathophysiology of OCD. These studies have contributed greatly to the development of neurocircuitry models of OCD, which emphasize the dysfunction of CSTC circuits in OCD patients [11,24,26,27]. The CSTC circuits have extensive connectivity to numerous cortical and subcortical regions, and the dysregulation of these connectivities within CSTC circuits are considered to be associated with OCD patients’ impaired executive performance [31], inability to inhibit cognitions and behaviors [32], and enhanced error monitoring processes [33,34]. Although the findings of structural neuroimaging studies have been relatively consistent with this view, findings from these studies have been inconsistent [48-50]. Confounding associated with psychotropic medication exposure may have contributed to the inconsistency across studies [18]. Using treatment-naïve OCD patients, our studies showed gray matter deficits in multiple regions of CSTC circuits, including increased regional gray matter volumes in the left thalamus and left caudate, as well as decreased regional gray matter volumes in the medial OFC and left ACC. Two recent meta-analyses of structural neuroimaging data revealed that OCD patients did show a reduced volume in the left ACC and OFC, as well as an increased volume in the thalamus and left caudate, which is highly consistent with our findings [9,51]. Among the CSTC circuits, some areas have been determined as “key brain regions”, including OFC, ACC, thalamus, and caudate nucleus. The OFC is believed to be involved in the process of decision making [52,53] and in inhibitory control of behavior [54,55]. In addition, the medial OFC has also been consistently identified as a component of DMN associated with the processing of risk and the inhibition of emotional responses [56]. Previous functional neuroimaging investigations found that the activity of OFC was increased in OCD patients during resting states [57,58], and was decreased after successful treatment [59], which suggests that this region may be involved in mediating the expression of OCD clinical symptoms. Several studies have proposed that OFC dysfunction in OCD might contribute to an overestimation of the risk that negative consequences may occur following a given action [16,60], and the impaired inhibitory processes that may be responsible for the repetitive behaviors observed in OCD [5]. Recent functional MRI studies have shown that the dysfunction of OFC may be also related to the disruption of reward learning in OCD patients [61]. The ACC is thought to be important in action-monitoring functions, including executive function, response selection, and conflict monitoring [34,62]. The aberrant structure in ACC found in this study may reflect dysfunction of action monitoring system and result in the abnormal symptoms of OCD such as the feelings of erroneous, constant need for correction and incomplete performance. The thalamus and caudate nucleus represent an important relay structure that transmits and processes neuronal information from the basal ganglia to cortical areas, and the dysfunction of thalamus and caudate nucleus are assumed to lead to implicit sequence learning and other important functions [63,64]. In addition, we also found that increased gray matter volume in the left thalamus was positively correlated with the total Y-BOCS scores of OCD patients, which is consistent with the findings of previous structural studies. Rotge et al. [65] reported that the severity of obsessive or compulsive symptoms correlated significantly with the effect sizes for the bilateral thalamus. These results suggest that thalamic volume is directly related to OCD severity. Taken together, our findings of structural abnormalities in CSTC circuits in OCD patients are consistent with those of a number of earlier structural, neuropsychological and functional studies, and further confirm that dysfunction of CSTC circuits may be at the root of the pathogenesis of OCD. 

In neuroimaging study on OCD, increasing attentions have now been paid to the DMN. The DMN is a prominent large-scale brain network characterized by a deactivation during goal-directed cognitive performance and increased activity in self-referential processing [66,67]. The function of the DMN has been implicated in attending to external and internal stimuli [68], as well as self-referential and reflective activity that specifically includes episodic memory retrieval, mental imagery, inner speech, and planning of future events [69,70]. The DMN consists of the medial prefrontal cortex, PCC, precuneus, inferior parietal lobules, parahippocampus, and some other brain regions [71]. The current findings reveal that OCD patients had aberrant gray matter volume in the medial OFC and PCC. The medial OFC is a part of the medial prefrontal cortex and is implicated in the processing of risk and fear. It also plays a role in the inhibition of emotional responses, and in the process of decision making [56]. Our structural data also show that structural abnormalities in the PCC play a role in the pathophysiology of OCD. Previous neuroimaging studies indicated that regional glucose metabolic rates and cerebral blood flow in the PCC could predict a subsequent response to treatment with psychotropic medication or cingulotomy [72,73]. There were several functional MRI studies using PCC as a seed region which showed DMN dysfunction among OCD patients [22,23]. However, until now there have been few reports of anatomic deficits in the DMN regions of OCD patients. Utilizing a whole-brain VBM analysis technique, the present study demonstrated that the PCC exhibited increased gray matter volume in treatment-naïve OCD patients. Dysfunction of DMN during the resting state is considered to be related to some OCD clinical symptoms such as the inability to get rid of persistent intrusive thoughts and images [22]. In that regard, our findings are consistent with functional MRI results, suggesting that anatomic deficits in the DMN regions (medial OFC and PCC) share an important function in the genesis or mediation of OCD symptoms. 

### The resting state functional connectivity analysis

There is increasing evidence that disrupted functional connectivity in OCD is considered to be a potential systems-level substrate of this disease. Functional MRI studies have reported alterations of functional connectivity in OCD patients and offered a series of meaningful information about the pathogenesis of OCD. Harrison et al. [24] used dorsal and ventral striatum (caudate and putamen) as the seed regions and found increased functional connectivity in the ventral striatum and OFC in OCD patients. Fitzgerald [27] assessed the functional connectivity of striatal and thalamic seed regions and showed altered functional connectivity within the CSTC circuits. Stern et al. [22] used anterior insular and several regions of DMN (PCC, medial frontal cortex, inferior parietal lobe, and parahippocampus) as seed regions and reported altered functional connectivity between the fronto-parietal network and the DMN. However, the seed regions used in the above functional studies were anatomic-integrated regions predefined subjectively by toolbox, such as WFU Pickatlas. In general, the pathogenesis of OCD is related to structural or functional abnormalities in some regions of the brain. The seed regions predefined subjectively in the above studies may not be the core hubs in the pathogenesis of OCD. In this study we used gray matter deficit regions based on our voxel-based morphometric results as the seed regions. This approach of functional connectivity analysis may have advantages over the above OCD functional connectivity studies based on a priori defined seed regions and provided more reasonable and persuasive findings of the pathogenesis of OCD.

In the present study, using gray matter deficit areas as seed regions in a cohort of treatment-naïve OCD patients, we found altered connectivity mainly in the CSTC circuits, DMN, and some other regions, including the cerebellum and temporal cortex. Relative to healthy controls, patients with OCD showed significantly increased connectivity within the CSTC circuits that included the bilateral lateral OFC, medial OFC, ACC, caudate, putamen thalamus, and left inferior frontal gyrus. We speculate that the OCD patients perhaps need to enhance the functional connectivity of these regions to compensate for their difficulty processing the alternating stimulus context, consistent with their phenomenological difficulty processing changing environmental contingencies and their overall cognitive inflexibility [74]. Most previous functional neuroimaging studies consistently showed increased brain activity in the OFC, caudate, and ACC in OCD patients compared to healthy controls [75-77]. Attenuation of abnormal regional functional activity within the CSTC circuits has been reported to be related to successful treatment with psychotropic medication or behavioral therapy in OCD patients [78-80]. Our findings, revealing functional deficits in CSTC circuits and default mode network, support and extend previous studies that have demonstrated the involvement of CSTC circuits and default mode network in the pathophysiology of OCD.

In the current study, we also revealed that the strength of functional connectivity within CSTC circuits was positively correlated with the total Y-BOCS scores of OCD patients, including the bilateral caudate, bilateral lateral OFC, medial OFC and right putamen. Previous neuroimaging studies reported that the metabolism in OFC of patients with OCD could predict the total Y-BOCS scores of patients with OCD [59,79]. Harrison et al. [24] showed the functional connectivity between ventral striatum and anterior OFC could be identified as a potential biomarker of symptom severity in OCD patients. Our earlier work also showed that the extent of spontaneous neuronal activity of the bilateral lateral OFC was positively correlated with the total Y-BOCS scores[15]. The current studies further provide strong evidence that dysfunction of CSTC circuits plays a very important role in the pathophysiology of OCD. Our findings added an expanding literature to the abnormality hypothesis of CSTC circuits by combining voxel-based morphometric and resting-state functional connectivity approach.

Several limitations should be considered when interpreting the results of the present study. First, the data are cross-sectional, therefore, the dynamic changes of brain structure and function during the progression of OCD could not be revealed by the current study. Future longitudinal studies can reveal deeper insight into the pathophysiology of OCD. Second, the OCD patients in the current study were heterogeneous in major symptom dimensions, and several structural and functional studies [17,26] showed that there are distinct neural substrates under different major symptom dimensions of OCD; however, we could not further divide the patients into different subgroups due to a limited sample size. Third, our use of global regression is a limitation in that there is continued controversy about the use of global signal regression in the preprocessing of resting state fMRI data. In this study, we regressed out the global signal to reduce the effects of the respiration [43,81]. While reanalyzing the fMRI data without whole-brain signal regression, our main findings of functional connectivity alterations in CSTC circuits and DMN were still observed. However, we also noticed that there were some differences in the results between the two different preprocessing choices. It indicates that the global signal might be physiologically meaningful, which needs to be careful to deal with in the future fMRI studies [82,83].

In conclusion, our findings provides evidence that morphologic and functional deficits are seen in CSTC circuits and default mode network in treatment-naïve OCD patients. At the same time, findings from the present study, along with future studies clarifying the causes of the morphologic and functional changes reported, may provide new insight into the underlying pathophysiology of OCD.

## Supporting Information

Figure S1 **Within-group functional connectivity maps of the healthy controls and OCD patients with and without global signal regression.** Maps threshold were set at *p*<0.05 with AlphaSim correction.
(TIF)

Figure S2 **Between-group differences in functional connectivity without global signal regression.** Maps threshold were set at *p*<0.05 with AlphaSim correction.
(TIF)

Figure S3 **Between-group differences in functional connectivity with correcting gray matter volume and global signal regression.** Maps threshold were set at p<0.05 with AlphaSim correction.
(TIF)
