# Supplementary figures and images for: Morphologic and Functional Connectivity Alterations of Corticostriatal and Default Mode Network in Treatment-Naïve Patients with Obsessive-Compulsive Disorder
Source: PLoS One. 2013 Dec 16;8(12):e83931. doi: 10.1371/journal.pone.0083931 (PMC3865285; doi:10.1371/journal.pone.0083931)

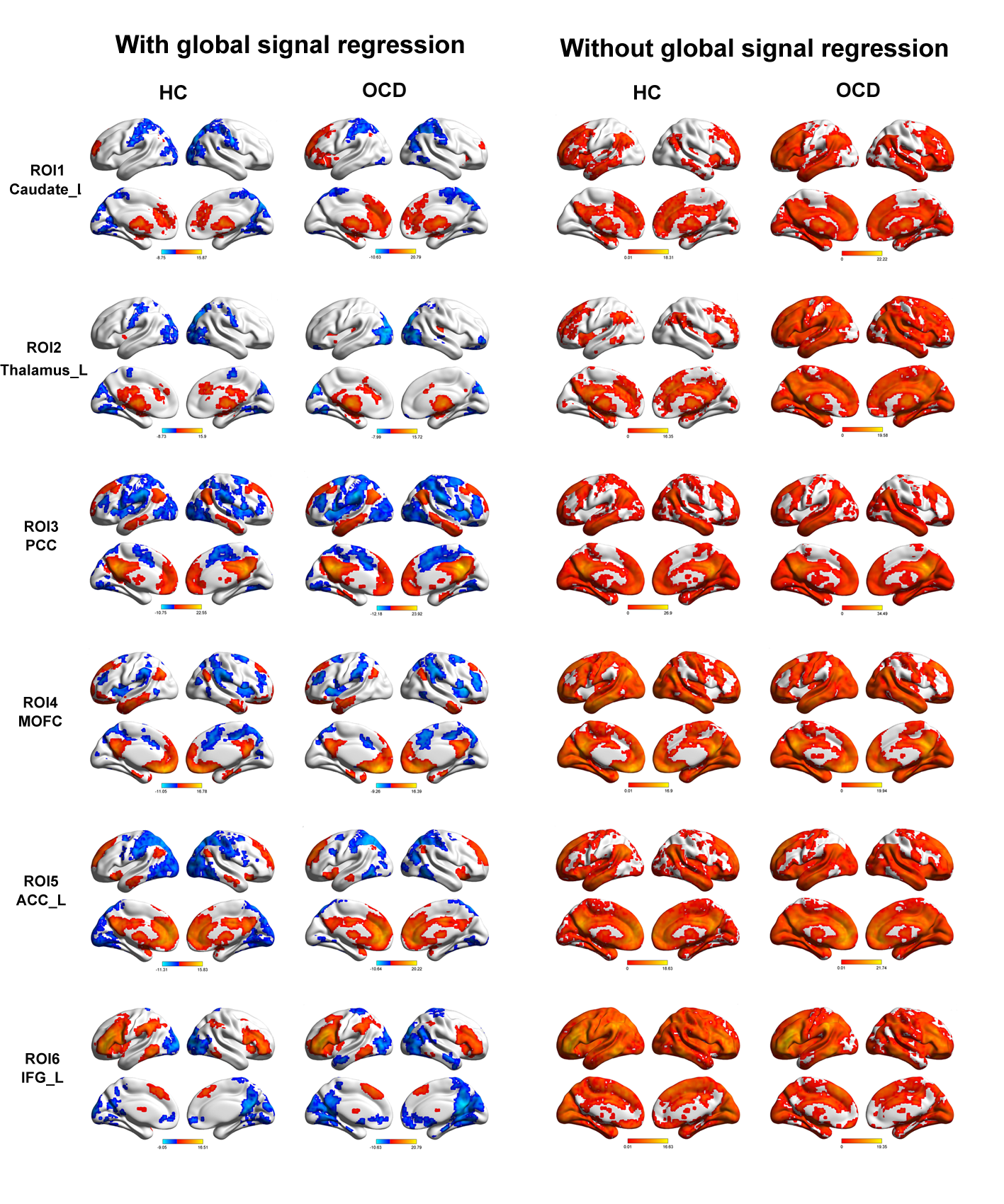

Supplement: Figure S1 — Within-group functional connectivity maps of the healthy controls and OCD patients with and without global signal regression. Maps threshold were set at p<0.05 with AlphaSim correction. (TIF) [file pone.0083931.s001.tif]

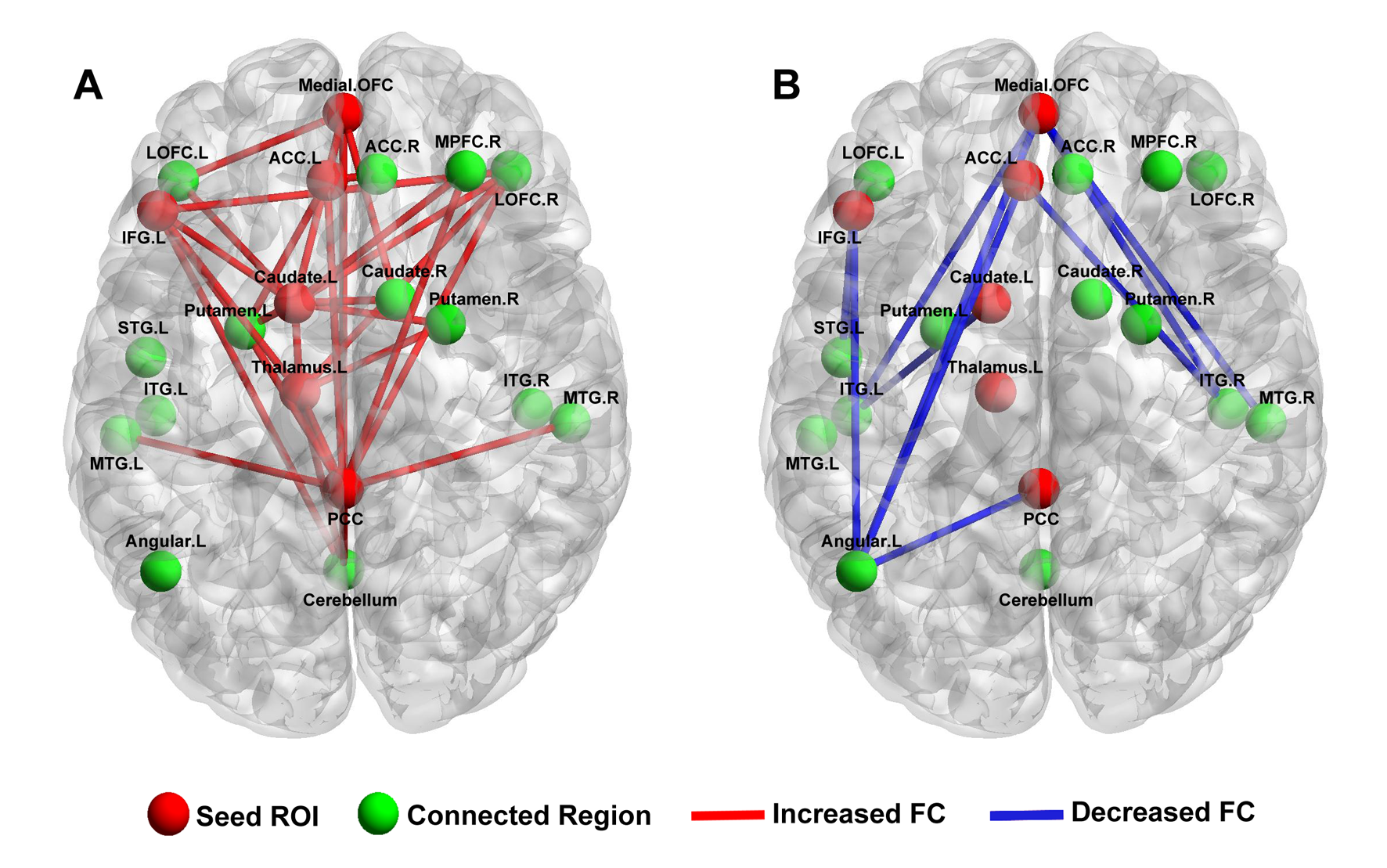

Supplement: Figure S2 — Between-group differences in functional connectivity without global signal regression. Maps threshold were set at p<0.05 with AlphaSim correction. (TIF) [file pone.0083931.s002.tif]

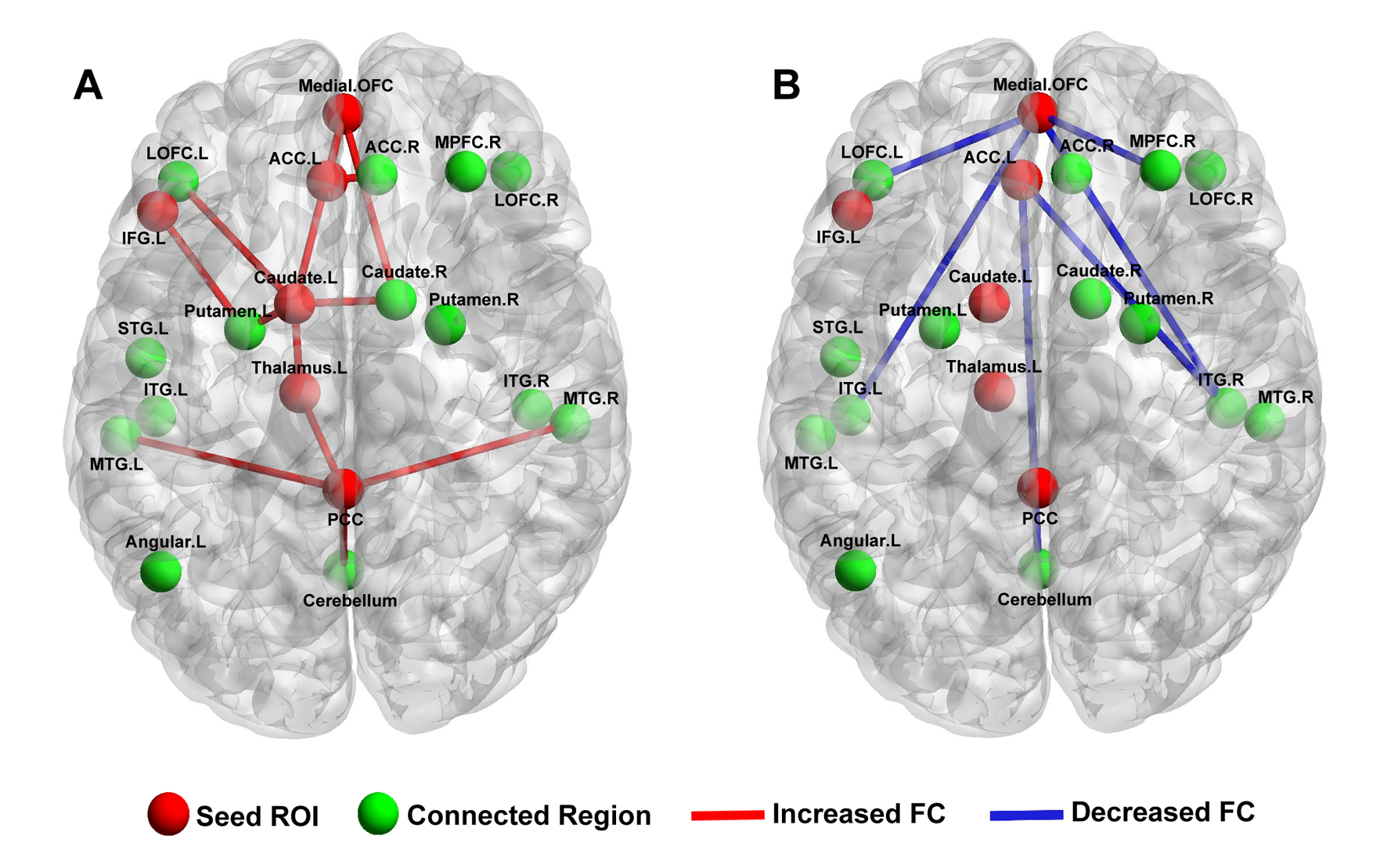

Supplement: Figure S3 — Between-group differences in functional connectivity with correcting gray matter volume and global signal regression. Maps threshold were set at p<0.05 with AlphaSim correction. (TIF) [file pone.0083931.s003.tif]
